# NEDA-state, psychological symptoms and quality of life are stable in natalizumab-treated multiple sclerosis patients: An up to 6-years long follow-up study

**DOI:** 10.1016/j.heliyon.2024.e39536

**Published:** 2024-10-18

**Authors:** Dániel Sandi, Zsófia Kokas, Zsigmond Tamás Kincses, Judit Füvesi, Zsanett Fricska-Nagy, Erika Vörös, Tamás Biernacki, László Vécsei, Péter Klivényi, Krisztina Bencsik

**Affiliations:** a Department of Neurology, Albert Szent-Györgyi Clinical Center and Faculty of Medicine, University of Szeged, Szeged, Hungary; b Department of Radiology, Albert Szent-Györgyi Clinical Center and Faculty of Medicine, University of Szeged, Szeged, Hungary; c ELKH-SZTE Neuroscience Research Group, University of Szeged, Szeged, Hungary

**Keywords:** Natalizumab, Multiple sclerosis, NEDA-3, Limb function, Pathopsychological symptoms, Quality of life

## Abstract

**Introduction:**

Natalizumab (NAT), a highly effective disease modifying therapy (DMT) in relapsing-remitting multiple sclerosis (RRMS), was approved for clinical use in Hungary on February 1, 2010. In this study we aimed to assess its effectiveness in view of the concept of “No Evidence of Disease Activity” (NEDA-3), furthermore evaluate its effect on limb function, pathopsychological symptoms (cognition, fatigue, depression) and quality of life (QoL).

**Patients and methods:**

From February 1, 2010, to December 1, 2022, 121 eligible patients were consecutively enrolled from the MS center of the University of Szeged, Hungary. Here, we report data on 6-years of follow-up. First, we evaluated the proportion of patients reaching the NEDA-3 state and any possible influencing factors. Then, we assessed the change of upper and lower limb functions via the 9-hole-peg test (9HPT) and the 25-feet walk test (TW25F). Finally, we assessed the change of pathopsychological symptoms (cognition, fatigue, depression) and QoL via the BICAMS, FIS, BDI-II and MSQoL-54 questionnaires, and the possible influencing factors behind it.

**Results:**

Cumulatively, 97 patients (80.2 %) achieved NEDA-3 throughout the follow-up period. On a year-by-year basis, the proportion changed from 95.9 % in the 1st year to 84.3 %, 81.3 %, 76.4 %, 74.5 % and 78.9 % in the 2^nd^, 3rd, 4^th^, 5^th^ and 6^th^ year respectively (p<0.001). Baseline EDSS scores and the type of preceding DMT affected this outcome. Both the upper and the lower limb functions remained stable. Cognitive functions improved (p<0.001), fatigue and depression scores remained stable during the follow-up period. QoL remained stable or improved in all subscales of MSQoL-54 questionnaire.

**Conclusion:**

Our 6-years long detailed follow-up study demonstrates that NAT not only reduces disease activity and progression. It effectively protects from the worsening of limb function, cognitive and other psychological impairments, and stabilizes the patients’ quality of life in basically every measurable aspect.

## 1 Introduction

Multiple Sclerosis (MS) is an autoimmune, demyelinating neurodegenerative disorder of the central nervous system (CNS). It usually affects young adults and is the leading cause of non-traumatic disability in this population [1]. Furthermore, it often leads to psychological disturbances (cognitive impairment, depression, anxiety and fatigue), all which strongly affect the patients' working capability, societal relationships and compliance, resulting in heavily decreased quality of life (QoL) [[2], [3], [4]]. Thus, timely diagnosis and treatment are of utmost importance to preserve the patients’ capabilities both in work and the everyday tasks.

The first therapeutic guideline of 2017 states that the main goal is the prompt and effective management of the disease to achieve the “no evidence of disease activity” (NEDA) state, defined as the combination of no new clinical relapse, no sustained disability progression and no MRI progression (new or enlarging T2-hyperintense lesion and/or Gadolinium (Gd)-enhancing T1-lesions) [5].

Natalizumab (NAT) was the first high efficacy disease modifying therapy (DMT) in relapsing-remitting MS (RRMS), first approved in 2004. It is a selective α4β1 adhesion molecule inhibitor and is considered to be among the most potent DMTs based on the phase III and the post-market observational phase IV trials. According to some evaluations, NAT also seems to have a beneficial effect on cognition and on QoL as well [6,7].

In the past 10–15 years large, long-term follow-up studies discussed the safety and efficacy of NAT (STRIVE, STRATA, TOP) [[8], [9], [10]]. However, only a few have been directly dedicated to assess the patients' NEDA-3 state or to evaluate NAT's effect on pathopsychological symptoms and QoL. Though the results were generally favorable, most of these studies were conducted on a low patient number with either short-term prospective follow-up or retrospective design.

In light of the aforementioned, the present study's primary objective was to determine efficacy of NAT in the real-world clinical practice by measuring the proportion of patients achieving NEDA-3 and the factors influencing it. We also aimed to analyze the reason behind not reaching the NEDA-3 state and to measure the proportion of patients with relapse-associated worsening (RAW) and progression independent of relapse activity (PIRA). Secondary endpoints were to assess the change in limb function during the follow-up period as measured by the timed 25 feet walk test (T25FWT) and the nine-hole peg test (9HPT). The tertiary objectives were to evaluate whether NAT has a beneficial effect on pathopsychological symptoms of the disease (cognition, fatigue and depressive symptoms) and the possible effect on self-reported QoL.

## 2 Patients and methods

### 2.1 Study design

This was a registry-based, single-center, single-arm, open-label, long-term follow-up study conducted completely in the Hungarian MS Center of Szeged.

Inclusion criteria were as follows.

1. Informed consent;
2. ≥18 years of age;
3. Definite diagnosis of RRMS according to the Poser, the original or the 2005, 2010 or 2017 revised McDonald criteria as applicable [[11], [12], [13], [14], [15]];
4. To have been prescribed natalizumab as part of the routine clinical practice and to remain on continuous NAT therapy for at least 1 year;
5. Baseline Expanded Disability Status Scale (EDSS) score of 0.0–6.5 points;
6. Known JC-virus (JCV) serostatus (since 2012).

Exclusion criteria were as follows.

1. Absence of informed consent;
2. Diagnosis of clinically isolated syndrome (CIS), primary progressive MS (PPMS), secondary progressive MS (SPMS);
3. Less than 1 year of continuous NAT therapy;
4. Baseline EDSS score >6.5 points.

The observation period began on February 1, 2010, and ended on December 1, 2022. Patients were enrolled consecutively and continuously. Due to statistical strength, here we report data up to 6 years of observation (mean duration: 3.5 ± 1.9 years). All patients received NAT with the standard, 4-week interval dosing. All data (sociodemographic, clinical, paraclinical) were derived from the MS Registry of Szeged where it was prospectively gathered. The registry is maintained since 1996, and was a paper-based database at the beginning, and was upgraded to a web-based electronic registry in 2013 [16]. The local ethics committee has approved this study (ethical approval number: 123/2013-SZTE).

### 2.2 Measures

Data collected before natalizumab initiation were age, age at disease onset, disease duration, relapses before NAT initiation, EDSS score before NAT initiation, previous DMTs, MRI at baseline, JCV status.

Data collected during NAT treatment were on-treatment relapses, EDSS score, T25FWT, 9HPT, follow-up MRI, JCV status and data on pathopsychological symptoms and QoL. For short cognitive screening, the paced auditory serial addition test (PASAT-3) and the symbol digit modalities test (SDMT) were used [17,18]. To evaluate depressive symptoms and fatigue, the Hungarian versions of Beck's Depression Inventory 2nd edition (BDI-II) and the fatigue impact scale (FIS) were utilized [19,20]. To measure QoL, the Hungarian version of the multiple sclerosis quality of life 54 (MSQoL-54) questionnaire was used [21]. The EDSS score, 9HPT, T25FWT, John Cunningham virus (JCV) status was recorded every 6 months. The PASAT, SDMT, FIS, BDI-II and MSQoL-54 data were recorded every 12 months.

Clinical relapse was defined according to the standard definition: the appearance of new, or worsening of already existing neurological symptoms, which lasted at least for 24 h, were not associated with fever or infectious disease and resolved within 3 months of onset in absence of corticosteroid therapy. Disability progression was defined as an increase of ≥1.5 points from an EDSS score of 0 point, ≥1 point from a baseline EDSS score of between 1.0 and 4.5, or ≥0.5 points from a baseline EDSS score ≥5.0 points. Disability progression was always confirmed 12 and 24 weeks after the initial assessment on a scheduled visit. MRI progression was defined as the appearance of one or more new, or the enlargement of one or more already existing T2-hyperintense lesion(s) or the appearance of one or more Gd-enhancing T1-lesion(s) on the subsequent follow-up MRI scans. All MRI scans were checked and validated by a neuroradiologist experienced in MS. NEDA-3 state was defined as the absence of clinical relapse, confirmed disability progression and MRI progression. RAW was defined as confirmed EDSS progression that happened during a clinically established relapse and fully or partially persisted 3 months after the event. PIRA was defined as confirmed EDSS worsening that happened without a clinical relapse or did not show any association with a previous relapse (i.e. happened at least after 3 months of a confirmed clinical relapse).

### 2.3 Statistical analysis

Categorical variables are presented as the number of cases and the proportion of cases in each category. In case of continuous variables, data are presented using either the mean and standard deviation (SD) or the median and inter-quartile range (IQR). Due to the non-normal distribution of data, non-parametrical tests were utilized. To measure the possible change through time of the observed parameters, Friedman's repeated measures ANOVA, Wilcoxon matched pairs test and Cochran's Q-test were used as was suitable. The chance of reaching NEDA-3 state was analyzed using the Cox proportional hazard model approach. General linear mixed models were utilized to analyze the possible factors influencing QoL and cognitive measures. In case of patient data were missing, the patient was lost to follow-up or stopped natalizumab therapy, data were taken into consideration up to the point of discontinuation. All analyses were performed using IBM's SPSS 24.0 software package.

## 3 Results

### 3.1 Baseline characteristics

During the observational period, 121 patients could be enrolled to the study. Of those patients, 102, 64, 55, 47 and 38 patients completed 2, 3, 4, 5 and 6 years of observation respectively. The mean follow-up duration was 3.5 ± 1.9 years with a median follow-up period of 3 years.

The cohort consisted of 94 women (77.7 %) and 27 men (22.3 %). The mean age of the patients was 44.9 ± 11.7 years. The mean age at disease onset was 28.9 ± 9.3 years, the mean disease duration was 15.8 ± 8.9 years. The median EDSS score at initiation was 2.0 points (IQR: 1,75). Dichotomized at the level of EDSS score 3 points, 93 patients (76.9 %) commenced NAT therapy at EDSS score 0–3 points, while 28 patients (23.1 %) NAT was initiated at EDSS score 3.5–6.5 points. High relapse activity, defined as either 2 relapse in the previous year or at least 3 relapses in the last 2 years before NAT initiation was found in 96 patients (79.3 %). Natalizumab was the first DMT for 19 (15.7 %). Of the 102 patients receiving any other DMT before natalizumab, 82 (80.4 %) were escalated from injectable therapies (interferon-β agents or glatiramer-acetate), while 20 patients (19.6 %) received oral agents (teriflunomide, dimethyl-fumarate or fingolimod) immediately before switching to NAT.

During the follow-up period, 46 of the 121 patients (38,0 %) stopped natalizumab treatment overall. The grand majority (35 patients; 76,1 %) chose to discontinue NAT due to high titer JCV positivity or seroconversion during the therapy, thus the elevated risk of progressive multifocal leukoencephalopathy (PML); 4 patients stopped the therapy due to inefficacy, of which 2 patients were proven to have NAT-neutralizing antibodies. One patient died during the follow-up up (committed suicide due to personal life crisis – no association with the therapy), one developed malignant melanoma thus NAT became contraindicated. Two patients converted into SPMS, thus NAT lost any indication. Overall, only 2 patients stopped NAT due to serious infectious side effects. Finally, 1 patient asked the therapy to be terminated due to self-reported subjective intolerance. We had no case of PML during the observational period.

### 3.2 NEDA-3, RAW and PIRA

**Fig. 1:**
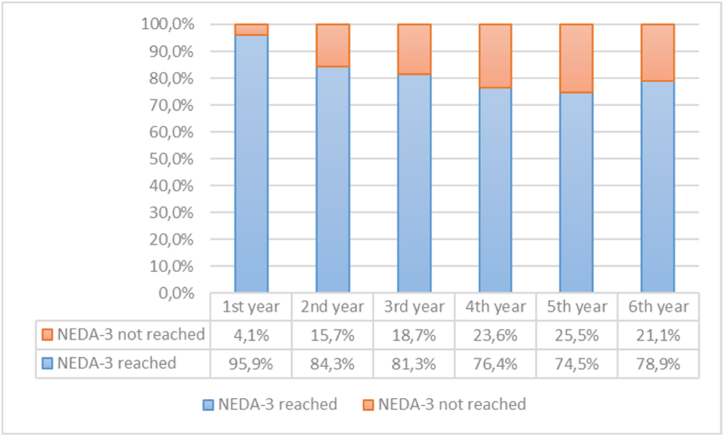
The proportion of patients reaching NEDA-3 state on a year-by-year basis.

During the follow-up period, 97 patients (80.2 %) achieved NEDA-3 cumulatively. On a year-by-year basis, the proportion of patients reaching the NEDA-3 state changed from 95.9 % in the 1^st^ year to 84.3 %, 81.3 %, 76.4 %, 74.5 % and 78.9 % in the 2^nd^, 3^rd^, 4^th^, 5^th^ and 6^th^ year respectively (Fig. 1). The change was significant only between the 1^st^ and 2^nd^ year (p<0.001).

Of the 24 patients, who did not reach the NEDA-3 state, 3 patients (12.5 %) experienced only clinical relapse; 2 patients (8.3 %) had only MRI progression, 10 patients (41.7 %) experienced only EDSS progression; 8 patients (33.3 %) had both EDSS progression and relapse, while 1 patient (4.2 %) experienced clinical relapses, EDSS progression and MRI progression as well.

In light of the concepts of RAW and PIRA, we found that 12 patients (50.0 %) experienced PIRA, 4 patients (16.7 %) experienced RAW, 3 patients (12.5 %) experienced both phenomenon, while 5 patients (20.8 %) had no EDSS progression whatsoever.

#### 3.2.1 Factors that influenced reaching the NEDA-3 state

**Fig. 2:**
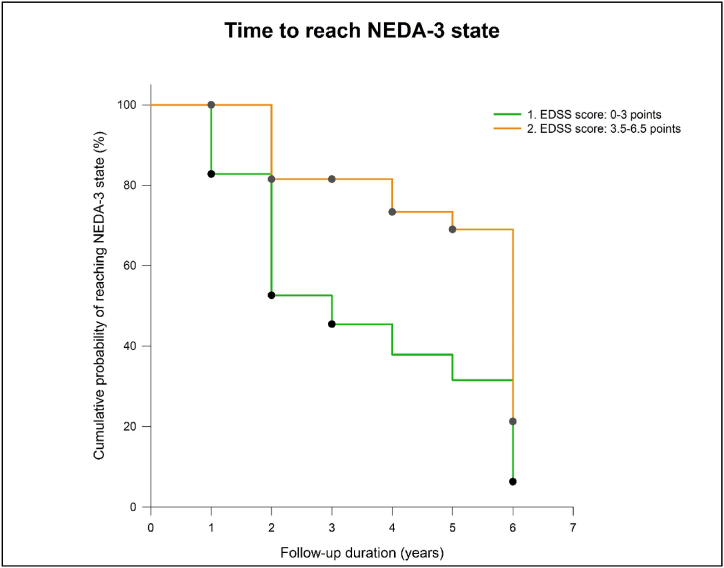
Time for patients to reach NEDA-3 state based on the baseline EDSS score.

The Cox proportional hazard model was utilized to assess the parameters that would affect reaching the NEDA-3 state. The assessed parameters were age, sex, disease duration, baseline EDSS score and the type of previous DMT. We determined that initial EDSS score and the type of preceding DMT therapy had a significant effect. Patients with an EDSS score of 0–3 points at NAT initiation had an almost twofold chance (HR: 2.03; 95 % CI: 1.20–3.443, p = 0.008) as compared to those patients, whose baseline EDSS scores were between 3.5 and 6.5 points (Fig. 2).

**Fig. 3:**
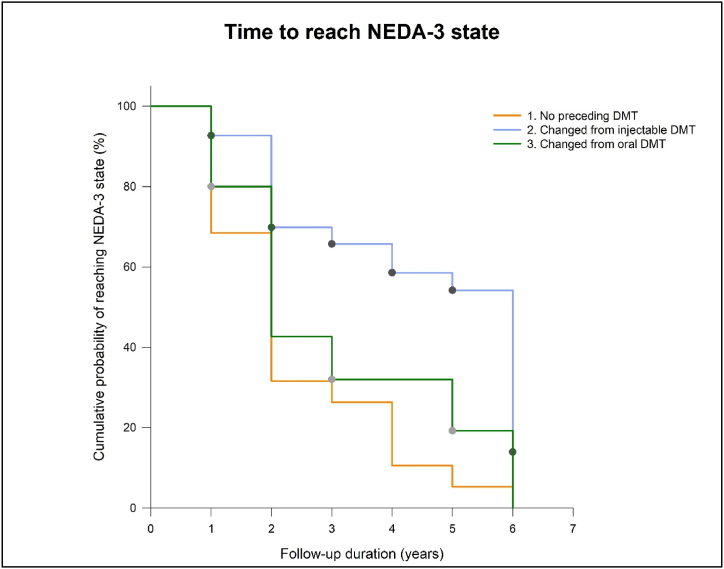
Time for patients to reach NEDA-3 state based on preceding DMT therapy.

Also, the type of preceding therapy significantly influenced the NEDA-3 status: patients, who received injectable therapies before NAT, had an 50 % less chance (HR: 0.48, 95 % CI: 0.28–0.85; p = 0.009) to maintain NEDA-3 state as compared to patients who received oral therapies or NAT was their initial DMT. However, there was no difference between the groups of therapy-naïve patients and patients whose preceding DMT was an oral agent (Fig. 3).

### 3.3 25-Feet walking test and the 9-hole peg test

During the follow-up period, no statistically significant change could be observed regarding the T25FWT (p = 0.302 and p = 0.668 in the first and second test respectively). Regarding the 9HPT performance of the patients, significant improvement could be observed in the first year of both the dominant and non-dominant hands performance during the first trials (p<0.001), however there was a slight worsening between the 5th and 6th year performance in case of the non-dominant hand (Table 1). There was no change regarding the second tests of neither hands (p = 0.425 and p = 0.148).

**Table 1:** The mean scores and the significance of change from baseline to 6th year during the first trials of the dominant and the non-dominant hand during the 9-hole peg test.

Time	Dominant hand		Non-dominant hand	
	Mean + SD	Significance (year-to-year)	Mean + SD	Significance (year-to-year)
*Baseline*	26.02 ± 6.40	Not applicable	28.18 ± 6.79	Not applicable
*1. year*	24.90 ± 6.94	<0.001∗	25.41 ± 5.82	<0.001∗
*2. year*	24.99 ± 6.40	0.877	25.84 ± 6.52	0.560
*3. year*	25.56 ± 8.22	0.436	26.24 ± 7.15	0.836
*4. year*	25.04 ± 7.55	0.881	24.95 ± 6.28	0.110
*5. year*	24.33 ± 7.31	0.096	24.91 ± 7.74	0.991
*6. year*	26.04 ± 11.28	0.161	26.01 ± 7.34	0.023∗

SD, standard deviation.

### 3.4 Cognitive performance, fatigue, depressive symptoms and quality of life

Due to missing data, only 4 years of follow-up could be analyzed in case of the SDMT, FIS, BDI and MSQoL-54 questionnaires. Concerning PASAT, 86, 78, 54 and 44 patients, in case of the SDMT 94, 83, 52 and 42 patients; regarding the FIS and BDI questionnaires 95, 87, 49, 44 and 104, 86, 52, 46 patients; and in case of the MSQoL-54 questionnaire 104, 87, 53 and 48 patients’ data could be analyzed in the 1^st^, 2nd, 3^rd^ and 4th years respectively. In the analysis, sex, age, disease duration, baseline EDSS score, preceding DMT, FIS and BDI scores were utilized as covariates.

During the 4-years follow-up, the PASAT scores significantly improved, which occurred in the course of the 1^st^ and the 3^rd^ year (Table 2). Based on the mixed models approach lower BDI scores (p = 0.017) resulted in better performance. Regarding SDMT, the mean scores significantly improved (p<0.001) during the follow-up, which was due to a significant improvement during the 1^st^ year (p<0.001) and the 2nd year (p = 0.019) (Table 2). Based on the mixed models approach, higher baseline EDSS score (p = 0.001), switching from injectable DMT (p = 0.003), higher BDI score (p = 0.003), higher FIS score (p = 0.004) led to worse performance.

**Table 2:** The mean scores and the significance of change from baseline to the 2nd and 3^rd^ year of the cognitive assessments PASAT and SDMT.

Time	SDMT		PASAT	
	Mean + SD	Significance (year-to-year)	Mean + SD	Significance (year-to-year)
*Baseline*	44.84 ± 14.03	Not applicable	44.48 ± 11.30	Not applicable
*1. year*	48.65 ± 11.45	<0.001∗	48.30 ± 11.26	<0.001∗
*2. year*	48.51 ± 13.39	0.019∗	49.16 ± 10.09	0.074
*3. year*	48.08 ± 14.96	0.861	51.52 ± 10.53	0.006∗
*4. year*	47.07 ± 14.00	0.084	53.20 ± 9.57	0.144

SDMT, Symbol Digit Modalities Test; PASAT, Paced Auditory Serial Addition Test; SD, standard deviation.

The patients’ FIS and BDI scores remained stable (p = 0.378 and p = 0.292 respectively) throughout the 2-years follow-up.

In the MSQoL-54 questionnaire, 13 of the 14 subscales remained unchanged during the 4-years observation. On the “Health distress” scale, a significant improvement could be observed globally, which can be attributed to the improvement of the scores in the 1^st^ year (Table 3). Based on the mixed models approach, the lower FIS (p<0.001) and BDI (p<0.001) scores resulted in less subjective distress about health for the patients.

**Table 3:** The mean scores and the significance of change from baseline to the 2nd year in the “social function” and the “health distress” subscales of the MSQoL-54 questionnaire.

Time	Health Distress	
	Mean + SD	Significance (year-to-year)
*Baseline*	54.49 ± 24.69	Not applicable
*1. year*	63.70 ± 24.06	<0.001∗
*2. year*	60.69 ± 25.60	0.761
*3. year*	66.37 ± 25.40	0.515
*4. year*	67.23 ± 24.71	0.638

SD, standard deviation.

## 4 Discussion

Natalizumab is a highly effective DMT in the management of RRMS patients according to both the initial clinical trials (AFFIRM, SENTINEL) and the ongoing long-term follow-up assessment (TOP) as well [6,7,9]. However, as our view on the management of patients evolves, real-world data are needed to provide further information and to aid in clinical decision making. Despite powerful data supporting the effectiveness of NAT, apart from TOP, long-term follow-up studies on NAT are limited. This is true even more so, when we consider emerging outcomes of everyday practice, such as limb-function tests, cognitive, other pathopsychological and QoL assessments. Data on these are relatively scarce and based on small, short, mainly retrospective assessments. Thus, in this study we aimed to present an analysis of the complex effect of NAT on the diverse aspects of MS.

In our cohort, almost 96 % of the patients maintained the NEDA-3 state at 1-year mark of the follow-up. However, this was significantly reduced in the 2nd year to 85 %, then some gradual small change could be observed which did not reach statistical significance. These results are somewhat controversial compared to the data in the literature. The 4-year long follow-up study (STRIVE), and a recent well-constructed analysis from the Czech Republic observed a reversed tendency. In STRIVE cumulatively 58 %, of the 4-year completers 65 % reached NEDA-3 state at the end of the observation, while the proportion was 56 % at 1^st^ year and 75 % in the 4th year [10]. In the Czech evaluation, approximately 52 % reached NEDA-3 state at the 1^st^ year mark, and 87 % in the 5th year of observation [22]. However other studies that compared the effectiveness of NAT to fingolimod (FIG) (alone or in combination with platform therapies) showed similar tendencies to our study, albeit with lower proportion of patients reaching the NEDA-3 state [[23], [24], [25]]. Another recent analysis from Switzerland, examining patients switching to FIG from NAT however showed, that approximately 90 % of the patients remaining on NAT reached NEDA-3 in every year constantly during the 5-year follow-up [26]. The explanation of these differences may partly lie in the differences of the examined populations. In the Italian examination comparing NAT to FIG, only non-responders to injectable therapies were included with higher baseline EDSS score and higher preceding ARR [23]. This might have accounted for the lower success rate as compared to our evaluation. In the Swiss examination however, patients receiving NAT both as first-line and second-line therapy were involved, with a similar level of baseline EDSS score, but with shorter disease duration and younger age [26]. These characteristics may have contributed to the exceptionally high proportion of patients reaching NEDA-3 state continuously. Most of the studies did not assess the probable risk factors for re-emerging disease activity, albeit we found that our patients who had higher EDSS scores at NAT initiation and those who changed from injectable DMTs had a worse prognosis for reaching the NEDA-3 state. Higher baseline EDSS has been associated with worse prognosis in many studies: both natural history or interventional, including a recent registry-based assessment regarding NAT from Austria [6,[27], [28], [29]]. The difference arising from the type of the preceding DMT (if there were any) is harder to explain however: patients previously receiving injectable DMTs had a significantly longer disease duration and switched DMTs more times previously, thus making these patients more susceptible for disease activity. This idea is reinforced by the recent TOPICS long-term assessment from Greece, showing that patients receiving no or maximum 1 prior DMT prior to NAT have a significantly higher chance of remaining free of relapse and EDSS progression as well [30]. However, more data is needed to draw a clear conclusion on the matter.

The failure to reach the NEDA-3 state was mainly driven by EDSS progression in our cohort. In the recent years it was shown, that this progression is usually not associated with clinical activity (relapse and/or MRI progression), thus the concept of PIRA arose, and can be used as the measure for “silent progression” or “smoldering MS” that challenges the dichotomy of “relapsing” and “progressive” MS [31]. We found, that in our study half of our patients who did not reach the NEDA-3 state experienced only PIRA, while in only 16.7 % of the cases progression could only be attributed to clinical activity, with also in case of another 12.5 % both phenomenon appeared. This is in line with data derived from the post-hoc analysis of the OPERA I and II clinical studies of ocrelizumab [31]. While the very low number of cases prevented us from making further on the matter, PIRA is an extremely important concept to investigate as it was shown when it appears early in the disease course, it highlights a worse prognosis for patients [32]. Thus, further analysis on its attributes and the effect of DMTs on it need to be conducted on large, population-based studies.

The secondary outcome measures of our study were measuring upper (9-HPT) and lower limb (T25FW) functions, which remained stable or even slightly improved in case of the hand performance. Data in the literature focusing on these functions are limited, the only two RCTs measuring these aspects were a retrospective analysis of the AFFIRM and SENTINEL studies measuring the effectiveness of the Multiple Sclerosis Functional Composite (MSFC), and the ASCEND study undertook on SPMS patients. However, both studies are randomized, placebo-controlled in nature, thus direct comparison with our results is not feasible. Regarding the first assessment, NAT significantly reduced the chance of progression on the limb functions [33]. In case of the second study, NAT had no positive effect on the lower limb function, however it was able to slow the progression in case of the 9-HPT [34]. These results combined with ours suggest, that NAT is able to stabilize limb functions long-term.

The tertiary outcome measures were the pathopsychological symptoms (cognitive performance, fatigue, depressive symptoms) and QoL measures. Both on PASAT-3 and on SDMT, our patients showed significant improvement during the 4-years follow-up. Data is somewhat conflicting in the literature on cognition regarding NAT. Some studies yielded negative results, however, most of these assessments agree, that NAT improves the cognitive performance of the patients [[35], [36], [37], [38], [39]]. However, the two longest follow-up investigations to date both yielded positive results similar to ours. Mattioli et al. found in their 3-years of follow-up showed a remarkably similar improvement in the patients PASAT-3 score [37]. Though in case of PASAT-3 practice/learning effect has been suggested to distort the results before, the fact that SDMT statistically improved as well makes it unlikely that learning effect played an important part in these results [33]. STRIVE, an ongoing multinational long-term follow-up study's recent results showed, that the SDMT performance of the patients improved during the follow-up period. More than 40 % of their patients demonstrated clinically meaningful improvement (i.e. ≥4 points change) that mainly occurred in the 1^st^ year of treatment, and the performance of the majority of these patients remained stable throughout the 4-years observation [40]. The very recent update from the Swedish MS Registry also prove, that cognitive performance improves after DMT initiation, and among all DMTs, the effect of NAT seems to be the largest [41]. All in all, these results suggest that NAT has a favorable effect on cognition. The performed regression analysis in our assessment identified several risk factors for the change in cognitive performance. Not surprisingly BDI was significant predictor for change in both tests, while baseline EDSS scores was a predictor in case of SDMT. These results are not surprising as both higher EDSS scores and moderate to severe depression has been linked to worse cognitive performance [[42], [43], [44], [45]]. However, in case of the SDMT, FIS performance and the type of preceding DMT also had an effect. On one hand, fatigue has been suggested to result in worse cognitive outcomes, however data in the literature are controversial at best regarding this matter, rarely finding significant associations [46,47]. Our results here imply connection between the two psychological symptoms, however more data is needed to draw any clear conclusions. In case of the type of preceding DMT, the changing from an injectable drug may suggest a more aggressive disease course and a possibly longer disease duration, which may explain these results, yet these explanations are speculative at best, thus these results need further evaluation.

FIS and BDI scores remained stable throughout the follow-up. Data regarding fatigue and depression during NAT treatment are even more scarce than data on cognition, with mixed results, which are mainly due to small sample sizes and different methodologies [[48], [49], [50]]. However, combining these data overall suggest that NAT has a positive effect on these symptoms as well.

QoL measures remained stable throughout the follow-up period, meaning that 13 of 14 MSQoL-54 subscales remained unchanged, while the “Health distress” scale showed significant improvement. The change occurred in the first year of treatment then scores remained stable in the subsequent years. In case of the health distress scale, we identified better FIS and BDI scores as significant predictors. These results are understandable, as fatigue and depression proved to be the most important predictors for QoL in MS according to recent studies, including our own analysis [3,51,52]. However, these observations are from transversal studies. Data regarding the longitudinal change of QoL in patients treated with NAT is scarce in the literature. A well-constructed study from the USA with more than 150 patients reaching 3 years of follow-up showed that QoL measures significantly improved from baseline and that patients with lower baseline EDSS score, shorter disease duration and younger age experienced larger improvement [53]. Another longitudinal study from France, albeit with a lower patient number (N = 48) also established, that patients’ QoL were either stable or improved during the 3 years of follow-up [48]. They identified the improvement of fatigue and depression to be two main contributors to change in QoL over the follow-up. Interestingly however, the French study found that patients with higher baseline fatigue and depression scores benefited more from the NAT therapy, which is in direct contrast with our results [48]. The aforementioned STRIVE assessment with high patient number also found QoL measures to be improved after 4-years of observation with implications that it may be connected partially to better cognitive performance [40]. All in all, the available results suggest, that QoL remains stable or even improves with NAT therapy, and the change in psychopathological symptoms plays an important role in this outcome.

There are some obvious limitations to our study. The relatively low number of patients and the single-center design provides less statistical power than a multi-centered study with a high number of participants. Also, the absence of a placebo group also makes direct comparison impossible. However, there are some strengths as well. The long-term follow up on a real-world cohort, and measuring infrequently evaluated, but highly important outcomes like NEDA-3 state, limb-function, pathopsychological symptoms and QoL provides a more complex view on the efficacy of NAT.

## 5 Conclusion

NAT has proven to be an effective DMT in RRMS patients in our 6-year longitudinal assessment. NAT does not only halt clinical and MRI activity, it significantly reduces disease progression, effectively protects from the worsening of limb function, cognitive and other psychological impairment, and stabilizes the patients’ quality of life in basically every measurable aspect as well.

## CRediT authorship contribution statement

**Dániel Sandi:** Data curation, Formal analysis, Funding acquisition, Investigation, Writing – original draft, Writing – review & editing. **Zsófia Kokas:** Data curation, Formal analysis, Writing – original draft, Writing – review & editing. **Zsigmond Tamás Kincses:** Data curation, Investigation, Writing – review & editing. **Judit Füvesi:** Data curation, Investigation, Writing – review & editing. **Zsanett Fricska-Nagy:** Data curation, Investigation, Writing – review & editing. **Erika Vörös:** Investigation, Writing – review & editing. **Tamás Biernacki:** Data curation, Investigation, Writing – review & editing. **László Vécsei:** Project administration, Supervision, Writing – review & editing. **Péter Klivényi:** Investigation, Supervision, Writing – review & editing. **Krisztina Bencsik:** Conceptualization, Data curation, Investigation, Methodology, Project administration, Resources, Supervision, Writing – original draft, Writing – review & editing.

## Data and code availability

The research data used in this article are not publicly available on legal and ethical grounds.

## Declaration of competing interest

The authors declare the following financial interests/personal relationships which may be considered as potential competing interests:Dr. Daniel Sandi reports financial support was provided by 10.13039/501100015763University of Szeged Open Access Fund (grant number 6050). If there are other authors, they declare that they have no known competing financial interests or personal relationships that could have appeared to influence the work reported in this paper.
